# Analysis of Peripapillary Retinal Nerve Fiber Layer Thickness in Acute Anterior Uveitis among Children with HLA-B27-Positive Juvenile Idiopathic Arthritis

**DOI:** 10.3390/jcm12144842

**Published:** 2023-07-22

**Authors:** Marta Świerczyńska, Agnieszka Tronina, Erita Filipek

**Affiliations:** 1 Department of Ophthalmology, Faculty of Medical Sciences in Katowice, Medical University of Silesia, 40-514 Katowice, Poland; 2 Department of Ophthalmology, Kornel Gibiński University Clinical Center, Medical University of Silesia, 40-514 Katowice, Poland; 3 Department of Pediatric Ophthalmology, Kornel Gibiński University Clinical Center, Medical University of Silesia, 40-514 Katowice, Poland

**Keywords:** anterior uveitis, juvenile idiopathic arthritis, enthesitis-related arthritis, HLA-B27, peripapillary retinal nerve fiber layer, pRNFL

## Abstract

Purpose: The aim of this study was to evaluate changes in the thickness of the peripapillary retinal nerve fiber layer (pRNFL) in children with a diagnosis of juvenile idiopathic arthritis (JIA) who were positive for human leukocyte antigen (HLA)-B27, treated for the first episode of unilateral acute anterior uveitis (AAU). Materials and Methods: This retrospective study included 41 children (aged 5 to 14 years; mean age 8.32 ± 2.4 years) with HLA-B27 positivity and unilateral JIA-AAU, and 40 healthy children. Optical coherence tomography (OCT) imaging was performed during active inflammation and subsequent noninflammatory phases (6 months after the resolution of inflammatory symptoms in the anterior segment of the eye). Results: There was a marked difference in mean pRNFL thickness between eyes with AU in the active phase, unaffected fellow eyes and the control group (110.22 ± 5.95 μm, 102.39 ± 4.39 μm and 95.83 ± 8.84 μm, respectively; *p* < 0.001). The thickness of pRNFL in eyes with AU in the active phase in all sectors was greater compared to unaffected fellow eyes (*p* < 0.001) and normal eyes (*p* < 0.001). In addition, it was demonstrated that pRNFL thickness was significantly increased in the superior and temporal sectors in the unaffected fellow eyes compared to the control group (128.73 ± 13.16 μm vs. 121.48 ± 13.35 μm and 71.37 ± 4.02 μm vs. 64.98 ± 9.12 μm, respectively). Even during the inactive phase, eyes with AU, compared to the healthy control group, had significantly greater pRNFL thickness in the inferior sector (129.78 ± 11.98 μm vs. 122.3 ± 14.59 μm; *p* = 0.018), along with the temporal sector (70.88 ± 5.48 μm vs. 64.98 ± 9.12 μm; *p* = 0.001). Conclusions: An increase in pRNFL thickness in children with unilateral JIA-AAU who were positive for HLA-B27 antigen can be observed in both eyes compared to healthy controls, and this change may persist even after the inflammatory symptoms have resolved. Measurements of pRNFL thickness resulting from JIA-AU-associated glaucoma should be performed during quiescent periods to avoid subclinical changes in pRNFL thickness caused by inflammation. However, when reviewing the results, it should be noted that changes in pRNFL parameters may be present despite evidence of a resolution of inflammation.

## 1. Introduction

Juvenile idiopathic arthritis (JIA) is a heterogeneous group of chronic arthritis conditions with a diverse clinical presentation, course and prognosis. JIA is defined as arthritis of not completely clarified etiology that develops in patients under the age of 16 years, persists for at least six weeks and for which other causes of arthritis have been excluded. It is the most common chronic rheumatologic disease of adolescence with an annual incidence of one to 22 cases per 100,000, having the lowest incidence in Asia and the highest in Scandinavian countries [1,2,3]. The prevalence rate ranges from seven to more than 400 cases per 100,000 [2,4]. There are seven subtypes of JIA based on the current classification according to the International League Against Rheumatism (ILAR) criteria [1]. Regarding JIA-associated uveitis (JIA-U), specifically anterior uveitis (AU), it is the most common extra-articular manifestation, affecting 11.6% to 30% of patients [5,6]. JIA-AU is defined in the oligoarticular and RF factor-negative category and is further distinguished by its chronic course, whereas acute AU (AAU) is most common in enthesitis-related arthritis (ERA) and associated with human leucocyte antigen (HLA)-B27 positivity [7]. 

It is estimated that glaucoma affects 25% of patients with childhood uveitis and is one of the main factors contributing to vision loss in its course [8]. There is twice that chance of an increase in intraocular pressure (IOP) in AU compared to intermediate or posterior uveitis, potentially due to the direct effect of inflammation on the trabecular meshwork [9]. Some evidence suggests that uveitis is more treatment-resistant in children and that children respond with an increase in IOP to steroid use twice as often as adults [10,11]. This might further be related to the fact that chronic uveitis is often asymptomatic and detected only at a stage when serious complications have already developed, resulting in treatment associated with prolonged use of topical and sometimes oral steroids [10]. Performing the Humphrey visual field test is often challenging, so in addition to IOP measurements, the only method to identify patients requiring more intensive treatment is the measurement of the peripapillary retinal nerve fiber layer (pRNFL). In fact, this can even indicate optic nerve damage earlier than visual field test and pinpoint those patients that require more intensive IOP-lowering treatment [12].

Previous studies have demonstrated that even mild AAU may result in changes in the posterior segment of the eye. However, these tend to remain subclinical and can be easily overlooked with biomicroscopy; nevertheless, the use of spectral domain optical coherence tomography (SD-OCT) can demonstrate the increase in central retinal thickness that develops with AUU [13,14,15,16,17,18,19]. The use of enhanced depth imaging OCT (EDI-OCT) has shown that choroidal thickness increases during the active phase of the disease and then decreases in response to treatment [18,20]. Using OCT angiography (OCT-A), it is noted that inflammation involving the anterior segment of the eye also affects the macular microvasculature [21]. Pre-existing research regarding the pRNFL likewise indicates that the thickness of the pRNFL increases, but these analyses have included adults with different AAU etiologies, and the control group has often consisted of the other eye of the same patient [18,22,23]. 

Therefore, the aim of the following study was to determine whether pRNFL thickness, assessed by SD-OCT, differs significantly between the active phase in the first episode of unilateral AAU and the inactive phase (6 months after the resolution of symptoms) in patients with JIA who were positive for HLA-B27 antigen. The results obtained in the study group were compared with pRNFL values from the fellow eye and healthy eyes from the control group. 

## 2. Methods

### 2.1. Study Population

We retrospectively analyzed the medical records of both the Pediatric Ophthalmology Outpatient Clinic and the Pediatric Ophthalmology Department, University Clinical Center in Katowice, Poland, from 2015 to 2022. The study included 41 Caucasian patients (82 eyes) treated for the first time for noninfectious unilateral AAU, who had previously been diagnosed with JIA and were positive for HLA-B27 antigen or were diagnosed during subsequent follow-up. The control group consisted of 40 patients (40 eyes) who were healthy children, matched for age and gender and routinely examined in the Pediatric Ophthalmology Outpatient Clinic. The study followed the principles of the Declaration of Helsinki.

### 2.2. Inclusion Criteria

The inclusion criteria for the study group were as follows:

(1) Aged ≥5 years and ≤16 years;
(2) Diagnosed with JIA;
(3) Positive for HLA-B27 antigen;
(4) A first episode of unilateral AAU diagnosed by the presence of inflammatory cells in the anterior chamber—grade ≥1+ (according to the Standardization of Uveitis Nomenclature classification [24]) and the absence of posterior vitreous cells;
(5) More than 5 days from onset of symptoms to OCT examination;
(6) Duration of AAU treatment ≤ 28 days;
(7) Remaining in the inactive phase of the disease for 6 months, which is defined as the absence of cells or haze in the anterior chamber;
(8) Spherical equivalent between −5.0 and +5.0 diopters (D);
(9) Obtained a good quality OCT scan (signal strength ≥ 6).

### 2.3. Exclusion Criteria

The study excluded patients who had:

(1) Infectious or traumatic etiology of AAU;
(2) Bilateral uveitis or vitritis, pars planitis, posterior uveitis or panuveitis;
(3) IOP ≥ 21 mmHg reported in medical history or noted during the treatment process;
(4) Glaucoma;
(5) Cup-to-disc asymmetry > 0.2, rim thinning, notching, excavation or RNFL defect;
(6) Optic nerve head drusen;
(7) Anisometropia ≥ 3.0 diopters or amblyopia;
(8) Lack of translucency of the optical media as well as other ophthalmologic disorders;
(9) Previous ophthalmologic surgery;
(10) History of ocular or head trauma;
(11) Used topical, periocular or systemic steroids > 3 months, immunosuppression or biologic therapy before the first episode of AAU;
(12) Been born before the end of the 37th week of gestation and/or birth weight < 2500 g;
(13) Occurrence of congenital systemic infections or exposure to toxins;
(14) Neurological comorbidities or developmental delay;
(15) Lack of patient cooperation during examination or poor quality of OCT scans.

### 2.4. Examination

Best corrected visual acuity (BCVA) was assessed in all patients using the Snellen chart or Lea symbols (in patients not familiar with the alphabet). The BCVA value was converted to logarithm of minimum angle of resolution (LogMAR) for statistical analysis. IOP was measured with a Goldmann applanation tonometer or a non-contact tonometer (Topcon RM-800B autorefractometer, Topcon Corporation, Tokyo, Japan); the results were adjusted to the central corneal thickness values measured with a CASIA2 Swept Source anterior segment OCT scanner, (Tomey Corporation, Nagoya, Japan). A slit-lamp examination of the anterior segment of the eyes was performed, in which anterior chamber cells were graded from 0 to 4 (according to the Standardization of Uveitis Nomenclature (SUN) classification [24]). The refractive defect recorded as the spherical equivalent (SE) was additionally measured (Topcon RM-8000B autorefractometer) and the fundus was assessed after pharmacological mydriasis (triple application of 1% Tropicamide). Axial length (AL) was measured with an optical biometer (IOLMaster 500, Carl-Zeiss Meditec, Dublin, CA, USA). Following pupil dilation, OCT scans (OCT Cirrus 6000, Carl-Zeiss Meditec, Dublin, CA, USA) were obtained to assess RNFL thickness. We performed imaging using a 200 × 200 optic disc cube protocol (200 horizontal scan lines, each of 200 A-scans). RNFL measurement included four sectors (superior, temporal, inferior and nasal) along with its average thickness. OCT scans with a signal strength ≥ 6 (maximum = 10) were defined as acceptable. The subsequent examination was performed in the noninflammatory phase of the disease, 6 months after the inflammation had resolved. All OCT scans were taken by two specialists. In control group children, the results of the dominant eye examination were included in the analysis. To compensate for AL-related ocular magnification, the Littmann formula (t = p∙q∙s), modified by Bennet and later adopted by Kang et al. [25,26,27], was used. In this particular comparison, t is the actual size of the fundus, p is the magnification factor of the camera, q is the magnification ratio of the eye, and s is the measurement obtained during OCT examination. This study relied on the use of a Cirrus HD-OCT scanner, for which p is 3.382, and q can be calculated with the formula q = 0.01306 − (AL − 1.82) to obtain a reliable measurement of RNFL thickness.

### 2.5. Statistical Analysis

The statistical analysis was performed using STATISTICA 13.3 software (TIBCO Software Inc., Palo Alto, CA, USA). The Shapiro–Wilk test was used to assess the prevalence of a normal distribution among the study variables. The Chi-square test was used to evaluate the differences between groups in terms of gender. To compare variables (BCVA at baseline and after resolution of uveitis, IOP, SE, scan quality index, pRNFL thickness) between eyes with AAU and healthy controls, as well as unaffected fellow eyes and healthy controls, the Mann–Whitney test was performed. For comparisons of AL between eyes with AAU and healthy control eyes as well as unaffected fellow eyes and healthy controls, an unpaired *t*-test was performed. The Wilcoxon signed-rank test was used to compare variables between eyes with AAU and unaffected fellow eyes and to compare eyes in the active and inactive AU phase. A *p*-value < 0.05 was considered the level of statistical significance.

## 3. Results

### 3.1. Demographic and Clinical Description of Patients with AAU

This study included 41 children (58.5% girls) with unilateral JIA-AU aged 5 to 14 years with a mean age of 8.32 ± 2.4 years, and 40 healthy children (52.5% girls) aged 6 to 15 years with a mean age of 8.03 ± 1.66 years. Among children in the study group, 61% were diagnosed with oligoarticular JIA and 39% with ERA. The mean duration of AAU was 14.1 ± 5.53 days. The analysis included eyes with AAU in active inflammation, unaffected fellow eyes and healthy control eyes. The BCVA (logMAR) value in eyes with active inflammation was 0.21 ± 0.11 (range 0.0 to 0.6), and it was 0.09 ± 0.1 (range 0.0 to 0.4) 6 months after the symptoms resolved. BCVA values in fellow eyes and control eyes were 0.08 ± 0.11 (range 0.0 to 0.4) and 0.05 ± 0.08 (range 0 to 0.3), respectively. The IOP in the eyes with active AU (15.73 ± 3.39 mmHg) was significantly higher compared to healthy control eyes, which was 12.48 ± 2.85 mmHg. The mean SE was −1.72 ± 2.34 D (range −5.0 to 3.5) in the eyes with AAU; −1.8 ± 2.14 D in unaffected fellow eyes (range −5.0 to 3.75) and −1.6 ± 2.71 D in normal eyes (range −5.0 to 4.5). The study did not reveal statistically significant differences between the groups regarding AL. However, the signal strength of the OCT imaging was different between the eyes with active AU and the control group (7.1 ± 1.1 vs. 8.08 ± 1.25) (Table 1).

### 3.2. pRNFL Thickness during the Active Phase of AU

The average pRNFL thickness was 110.22 ± 5.95 μm in eyes with AU in the active phase, 102.39 ± 4.39 μm in unaffected fellow eyes and 95.83 ± 8.84 μm in normal eyes. The average pRNFL thickness and pRNFL thickness in all sectors were found to be increased compared to unaffected fellow eyes (*p* < 0.001) and normal eyes (*p* < 0.001). Similarly, the average pRNFL thickness and pRNFL thickness in the superior and temporal sectors of the unaffected fellow eyes were also considerably higher than in the control group (*p* < 0.001) (Table 2).

### 3.3. pRNFL Thickness during the Inactive Phase of AU

In the inactive phase, the average thickness of the pRNFL in eyes with AU was 97.85 ± 8.05 μm, while in unaffected fellow eyes it was 96.63 ± 5.86 μm, and there were no statistically significant differences in relation to the control group (95.83 ± 8.84 μm). However, a relatively greater thickness of pRNFL was found in the inferior and temporal quadrants compared to the control group, 129.78 ± 11.98 μm (*p* = 0.018) and 70.88 ± 5.48 μm (*p* = 0.001), respectively. Comparison of eyes with AU in the active and inactive phases, as well as unaffected fellow eyes in the active and inactive phases, showed that the pRNFL significantly reduced its thickness after the active phase of inflammation terminated (*p* < 0.001) (Table 3).

## 4. Discussion

Previous studies have suggested that uveitis of various etiologies can have an impact on pRNFL thickness [9,18,22,23,28]. Shulman et al. [18] observed that eyes with AAU have a thicker macula and pRNFL compared to healthy fellow eyes, but only nine patients of the 14 patients studied entered this analysis after inclusion criteria were considered. Lee et al. [22] demonstrated that macular and pRNFL thickness increases significantly during the active phase of AAU and subsequently, during the inactive phase, decreases to levels that are equivalent to fellow eyes. Moreover, they found that pRNFL thickness showed a highly adequate response to the degree of inflammatory changes, and this measurement could potentially be useful when assessing disease activity. In a study among children with chronic nonjuvenile idiopathic arthritis and uveitis, it was found that during active inflammation, OCT scans demonstrated increased pRNFL thickness compared to quiescent and control eyes. Additionally, the thickening during AU was demonstrated to be less pronounced compared to intermediate uveitis. It was also indicated that there is significant thinning of the pRNFL in the lower sector in glaucomatous eyes compared to normotensive uveitis eyes [28]. Similarly, a retrospective study involving 309 patients with uveitis suggested that pRNFL thinning in the inferior quadrant may suggest glaucomatous changes in eyes with uveitis [9]. In contrast, an analysis that included different types of uveitis demonstrated that mean pRNFL thickness, as well as mean sectoral measures, excluding the superonasal part, were significantly increased in eyes with active uveitis compared to eyes with quiescent uveitis. The review study of groups with active uveitis and with and without glaucoma demonstrated a limited increase in pRNFL thickness in the former, suggesting that pRNFL atrophy in the course of glaucoma may limit pRNFL thickening [23]. It was also reflected in our study, in which children with unilateral JIA-AAU had a significant increase in mean pRNFL thickness compared to healthy control eyes, and the difference was evident in all sectors of the pRNFL.

The shift in pRNFL thickness in the course of AAU may be related to the release of inflammatory mediators capable of diffusing from the anterior to the posterior segment of the eye and triggering the destruction of blood–retinal barriers (BRB). High levels of vascular endothelial growth factor (VEGF), interleukin (IL)-6 and IL-1β, which increase vascular permeability, appear to have a significant effect [29]. An increase in pRNFL thickness has also been found after uncomplicated cataract or anti-glaucoma surgery, probably due to mild uveitis occurring in the postoperative period [30,31]. With OCT-A, an increase in vascular density was demonstrated in both the superficial and deep capillary plexus during the active phase of AAU, as well as a decrease in the area of the deep foveal avascular zone, although these changes resolved after the inflammation regressed. These studies emphasize the fact that regardless of anatomical location, uveitis can lead to microvascular changes [21]. Similarly, such changes can develop in the peripapillary area that features numerous vessels covering the entire retina.

Glaucoma associated with uveitis is the third most common (after cataracts and macular edema) cause of vision loss, being irreversible in contrast to the latter [32]. In the majority of eyes with JIA-AU, an increase in IOP has been detected once inflammation has become inactive (mean 4.5 ± 5 months), apparently as a result of normal ciliary body function as well as reduced uveoscleral and trabecular outflow rates caused by morphological changes [33]. Therefore, it is essential to carry out regular IOP measurements not only in the active, but also in the inactive phase of AU. Considering the previously described changes, screening for pRNFL thinning due to glaucoma should be performed with great caution in patients with JIA, as the results can be misleading. Cirrus HD-OCT shows excellent repeatability, and when the two measurements obtained from the same eye at two distinct visits are compared, the mean difference in pRNFL thickness ≥ 4 μm can be considered as statistically significant [34]. However, if uveitis and glaucoma coexist, a pRNFL thickness that is within normal limits may lead to less aggressive IOP-lowering therapy. On the other hand, if the pRNFL decreases as the inflammation resolves, this may lead to overly rapid confirmation of the progression of glaucomatous damage. Similarly, assessment of the appearance of the optic nerve discs can be difficult due to subclinical swelling of the pRNFL which can then mimic its normal appearance, with the cupping revealing itself only after the inflammation has subsided [35]. Interestingly, Kriegel et al. [36] found that active inflammation does not affect the measurement of Bruch’s membrane opening minimum rim width (BMO-MRW), so this parameter may become much more useful in detecting early glaucomatous changes during uveitis.

According to our study, there was also a significant increase in pRNFL thickness in unaffected fellow eyes (superior and temporal sectors) compared to healthy control eyes. To date, only a retrospective study by Lee et al. [22] conducted a similar comparison between adults with unilateral noninfectious AAU and the normal control group and found no significant differences. In contrast, Wexler et al. [13] demonstrated that subclinical macular thickening was present in AU-affected eyes as well as in unaffected fellow eyes of patients compared to healthy controls, and significantly greater macular thickness was noted in HLA-B27-positive compared to HLA-B27-negative patients. Similarly, Power et al. [14] found that cystoid macular edema during AU was five times more common in HLA-B27-positive patients than in HLA-B27-negative patients. However, in a study evaluating retinal thickening in HLA-B27-positive and -negative patients during iridocyclitis, no such relationship was observed [15]. These changes observed in unaffected fellow eyes can be explained to a certain degree by the finding that the peripheral blood of patients with uveitis had elevated levels of IL-22, which can disrupt the BRB and allow biologically active components and water to reach the retina. HLA-B27 homodimers on the surface of T cells, monocytes and natural killer (NK) cells trigger innate immune mechanisms by binding to killer immunoglobulin-like receptors (KIRs) [37], and increased expression of the KIR3DL2 receptor on the surface of NK cells in peripheral blood has been detected in AAU and HLA-B27-positive patients [38].

Evaluation of the progression of retinal thickening over time in patients with HLA-B27-associated AAU has demonstrated the persistence of minimal subretinal thickening over a period of 3 months [16]. Lee et al. [22] also noted that several patients continued to have a change in macular thickness compared to the unaffected fellow eye 3 months after the resolution of anterior segment symptoms, which the authors explained as due to insufficient recovery time. A study by Traill et al. [17] demonstrated that in a group consisting mainly of HLA-B27-positive patients, only 55% showed resolution of macular thickening after 6 months, regardless of resolution of AAU symptoms. However, it should be considered that this study included only patients with moderate-to-severe AAU. [39]. Moreover, ultrabiomicroscopy of the ciliary body can detect inflammatory changes 6 weeks after AAU disappears. Increased pRNFL thickness compared to healthy control eyes persisted in some patients in our study (inferior and temporal sectors) even 6 months after AAU symptoms had resolved. Unlike trauma, which triggers a transient inflammatory response, JIA-AU may be associated with chronic release of inflammatory mediators.

Limitations of our study, due to its retrospective nature, include the lack of OCT scan sequences assessing at which stage of AAU the increase in pRNFL thickness appeared and its progression over time. Evaluation of the ganglion cell complex (GCC) and measurement of inflammatory cells in the anterior chamber using the Laser Flare Meter may be beneficial; similarly, analysis of additional immunological parameters, such as antinuclear antibodies (ANA) or other HLA subtypes, could provide important information. A prospective study on a larger group of patients, with a higher frequency of OCT imaging scans and longer follow-up, is desirable. Nevertheless, this is the first study to analyze pRNFL in affected and normal fellow eyes, compared with healthy controls, in active and inactive phases among children with JIA-AAU and positive HLA-B27.

## 5. Conclusions

An increase in pRNFL thickness in children with JIA-AAU and who are positive for HLA-B27 can be observed in both eyes compared to healthy controls, and this change can persist even after the inflammatory symptoms in the anterior segment of the eye have resolved.

Among patients with JIA-AAU, secondary open-angle glaucoma and ocular hypertension often develop during the clinical course of the disease. Appropriate topical antiglaucoma treatment should be initiated immediately after diagnosis to prevent the development of irreversible optic neuropathy. Measurements of pRNFL thickness performed in patients with glaucoma associated with JIA-AU can be challenging due to the young age of patients. Moreover, during the active phase of AU, a normal-looking RNFL thickness may prompt the clinician to lower the IOP less aggressively. Measurement of pRNFL should be performed during quiescent periods to avoid the subclinical changes in pRNFL thickness caused by inflammation. However, when reviewing the results, it should be noted that changes in pRNFL parameters may be present despite the resolution of inflammation.

## Figures and Tables

**Table 1:** Demographic and clinical description of patients with unilateral acute anterior uveitis and children from control group.

	Eyes with AAU (41 Eyes) (Mean ± SD)	Unaffected Fellow Eyes (41 Eyes) (Mean ± SD)	Healthy Controls (40 Eyes) (Mean ± SD)	*p*1-Value ^†^	*p*2-Value ^§^
Age (years)	8.32 ± 2.4		8.03 ± 1.66	-	0.89
Female (*n* %)	24 (58.5)		21 (52.5)	-	0.58 ^#^
BCVA at initial examination (LogMAR)	0.21 ± 0.11	0.08 ± 0.11	0.05 ± 0.08	<0.001 *	0.23
BCVA after resolution of uveitis (LogMAR)	0.09 ± 0.09	0.07 ± 0.1		0.09	0.34
IOP (mmHg)	15.73 ± 3.39	13.93 ± 3.84	12.48 ± 2.85	<0.001 *	0.06
SE (D)	−1.72 ± 2.34	−1.8 ± 2.14	−1.63 ± 2.71	0.93	0.9
Axial length (mm)	24.15 ± 1.61	24.28 ± 1.66	23.98 ± 1.77	0.66 ^‡^	0.44 ^õ^
Mean duration of episode of uveitis (days)	14.1 ± 5.53			-	-
Scan quality index	7.1 ± 1.1	7.73 ± 1.16	8.08 ± 1.25	<0.001 *	0.24

AAU: acute anterior uveitis; BCVA: best-corrected visual acuity; D: diopters; LogMAR: logarithm of the minimum angle of resolution; IOP: intraocular pressure; SD: standard deviation; SE: spherical equivalence; * statistically significant difference (*p* < 0.05). ^#^ Comparison was performed using chi square test. ^†^ Comparison was performed using Mann–Whitney test between eyes with acute anterior uveitis and age-matched normal eyes. ^‡^ Comparison was performed using an unpaired *t*-test between eyes with acute anterior uveitis and age-matched normal eyes. ^§^ Comparison was performed using Mann–Whitney test between eyes with unaffected fellow eye and age-matched normal eyes. ^õ^ Comparison was performed using an unpaired *t*-test test between eyes with unaffected fellow eye and age-matched normal eyes.

**Table 2:** Comparison of the pRNFL thickness between eyes with anterior uveitis, unaffected fellow eyes and age-matched eyes from control group during the active phase of inflammation.

pRNFL Thickness (μm)	Eyes with AU (41 Eyes) (Mean ± SD)	Unaffected Fellow Eyes (41 Eyes) (Mean ± SD)	Healthy Controls (40 Eyes) (Mean ± SD)	*P*1-Value ^†^	*P*2-Value ^§^	*P*3-Value ^‡^
Average	110.22 ± 5.95	102.39 ± 4.39	95.83 ± 8.84	<0.001 *	<0.001 *	<0.001 *
Superior	137.29 ± 11.19	128.73 ± 13.16	121.48 ± 13.35	<0.001 *	0.008 *	<0.001 *
Nasal	80.54 ± 8.36	71.39 ± 7.6	70.13 ± 11.61	<0.001 *	0.42	<0.001 *
Inferior	140.24 ± 10.16	127.71 ± 10.86	122.3 ± 14.59	<0.001 *	0.1	<0.001 *
Temporal	81.29 ± 4.73	71.37 ± 4.02	64.98 ± 9.12	<0.001 *	<0.001 *	<0.001 *

AU: anterior uveitis; pRNFL: peripapillary retinal nerve fiber layer; SD: standard deviation; * statistically significant difference (*p* < 0.05). ^†^ Comparison was performed using Mann–Whitney test between eyes with anterior uveitis (in active phase) and age-matched normal eyes. ^§^ Comparison was performed using Mann–Whitney test between unaffected fellow eyes (during the active phase of AU) and age-matched normal eyes. ^‡^ Comparison was performed using a Wilcoxon signed-rank test between eyes with anterior uveitis (in active phase) and unaffected fellow eyes (during the active phase of AU).

**Table 3:** Comparison of the pRNFL thickness between eyes with anterior uveitis, unaffected fellow eyes and age matched eyes from control group during the inactive phase of inflammation (6 months after the resolution of symptoms).

pRNFL Thickness (μm)	Eyes with AU (41 Eyes) (Mean ± SD)	Unaffected Fellow Eyes (41 Eyes) (Mean ± SD)	*p*1-Value ^†^	*p*2-Value ^§^	*p*3-Value ^‡^	*p*4-Value ^õ^	*p*5-Value ^”^
Average	97.85 ± 8.05	96.63 ± 5.86	0.118	0.657	<0.001 *	<0.001 *	<0.001 *
Superior	123.59 ± 12.4	120.17 ± 12.2	0.379	0.861	<0.001 *	<0.001 *	<0.001 *
Nasal	70.02 ± 5.14	69.98 ± 8.6	0.737	0.629	<0.001 *	<0.001 *	<0.001 *
Inferior	129.78 ± 11.98	120.71 ± 10.87	0.018*	0.667	<0.001 *	<0.001 *	<0.001 *
Temporal	70.88 ± 5.48	67.17 ± 4.94	0.001*	0.091	<0.001 *	<0.001 *	<0.001 *

AU: anterior uveitis; pRNFL: peripapillary retinal nerve fiber layer; SD: standard deviation; * statistically significant difference (*p* < 0.05); ^†^ Comparison was performed using Mann–Whitney test between eyes with anterior uveitis (in inactive phase) and age-matched normal eyes. ^§^ Comparison was performed using Mann–Whitney test between unaffected fellow eyes (during inactive phase of AU) and age-matched normal eyes. ^‡^ Comparison was performed using a Wilcoxon signed-rank test between eyes with anterior uveitis (in active phase) and unaffected fellow eyes (during active phase of AU). ^õ^ Comparison was performed using a Wilcoxon signed-rank test between eyes with AU in active and inactive phase. **^”^** Comparison was performed using a Wilcoxon signed-rank test between unaffected eyes with AU in active and inactive phase.

## Data Availability

The dataset analyzed in this study is available from the corresponding author upon reasonable request.
